# Supplementary material for: Analysis of chi angle distributions in free amino acids via multiplet fitting of proton scalar couplings
Source: Magn Reson (Gott). 2024 Aug 19;5(2):103–20. doi: 10.5194/mr-5-103-2024 (PMC11570886; doi:10.5194/mr-5-103-2024)
Supplement: The supplement related to this article is available online at: https://doi.org/10.5194/mr-5-103-2024-supplement. [file mr-5-103-2024-supplement.zip › Syed2024/data/fit1d_fitnmr_output.tar.gz!/fit1d/arg/500/bmse000029_simulation_1_nmredata/1H/resonances_1d.pdf]

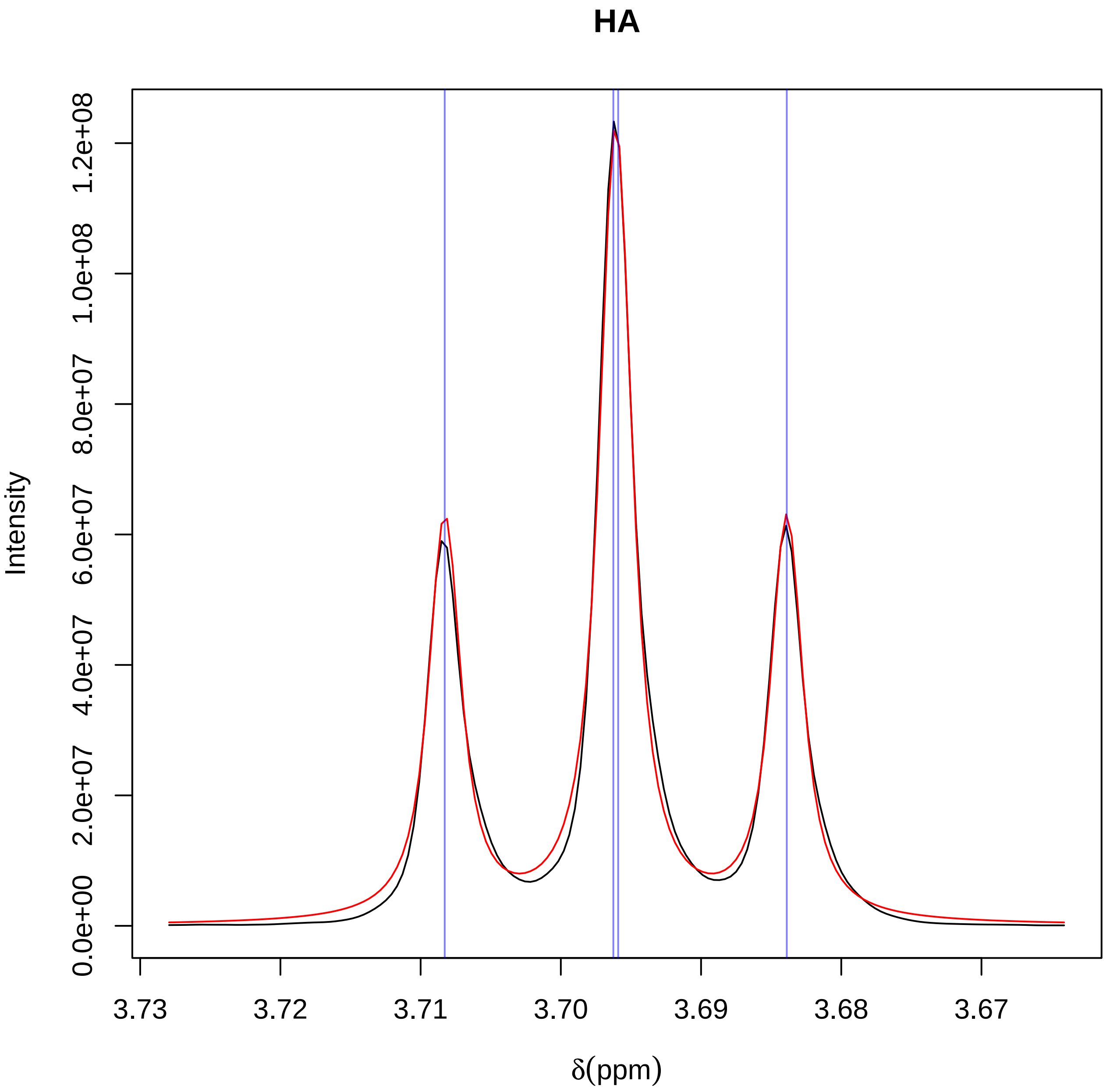

HA
Intensity
0.0e+00
2.0e+07
4.0e+07
6.0e+07
8.0e+07
1.0e+08
1.2e+08
3.73
3.72
3.71
3.70
3.69
3.68
3.67
δ(ppm)

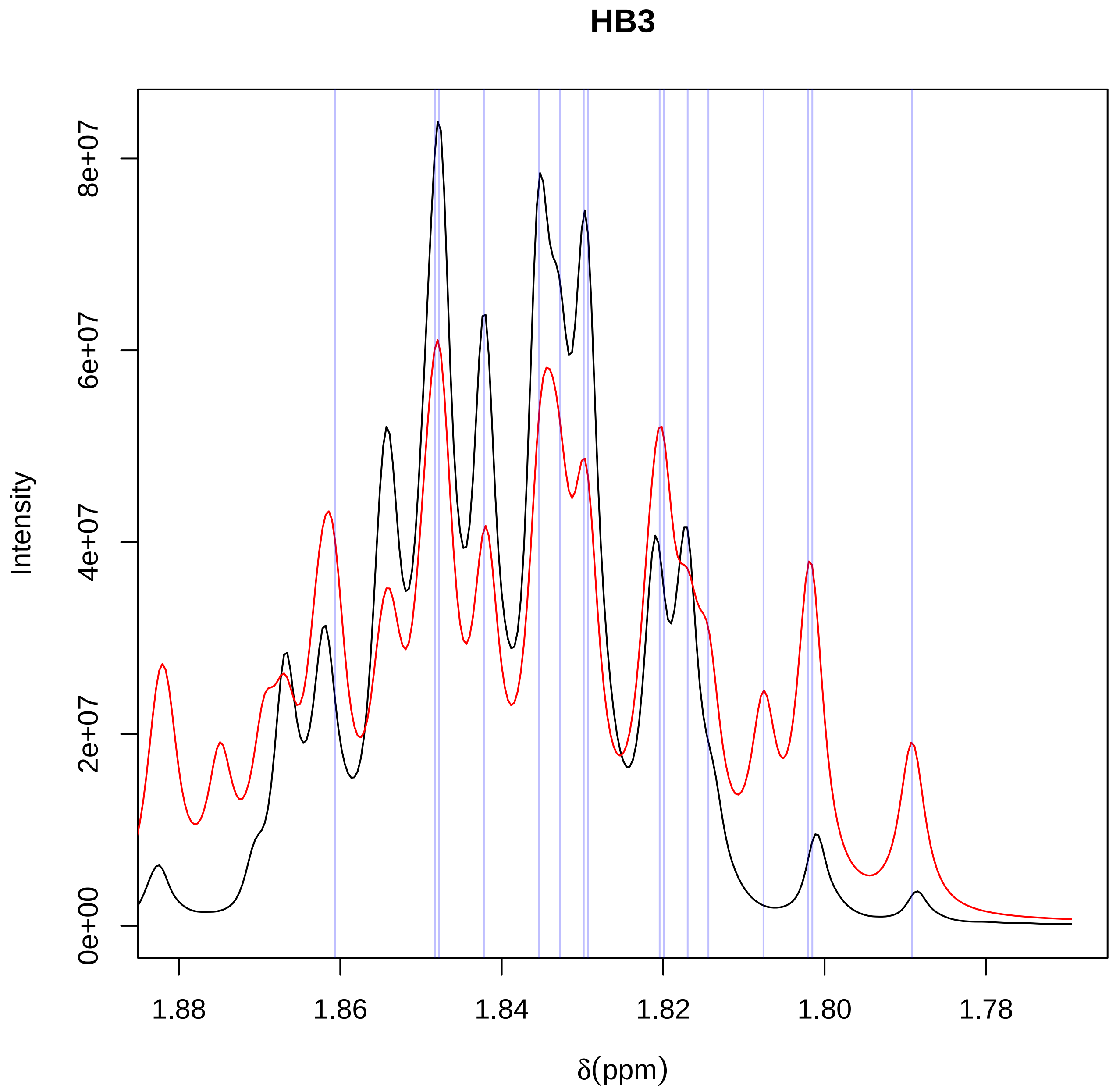

HB3
Intensity
0e+00
2e+07
4e+07
6e+07
8e+07
1.88
1.86
1.84
1.82
1.80
1.78
δ(ppm)

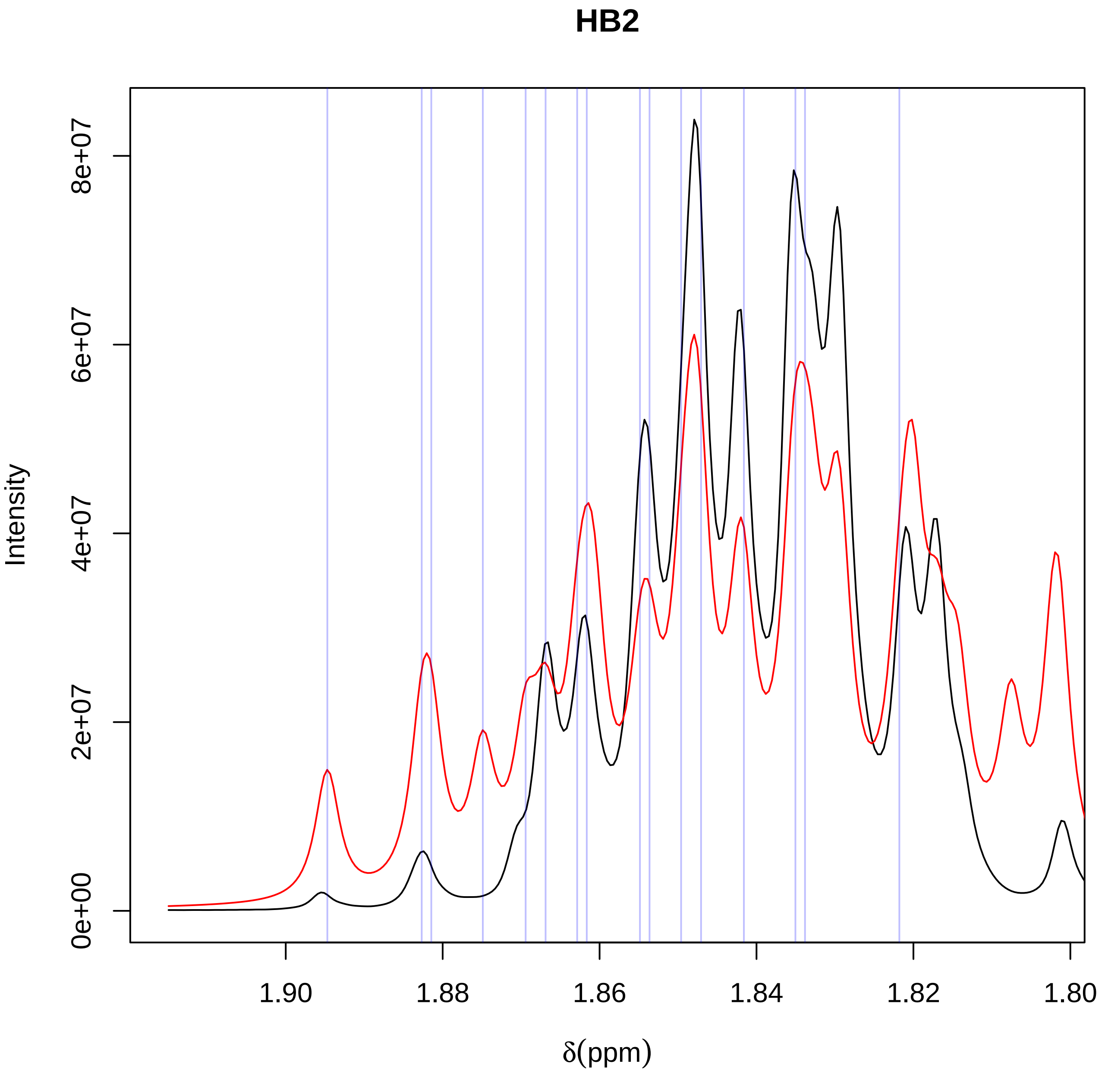
HB2
Intensity
0e+00
2e+07
4e+07
6e+07
8e+07
1.90
1.88
1.86
1.84
1.82
1.80
δ(ppm)

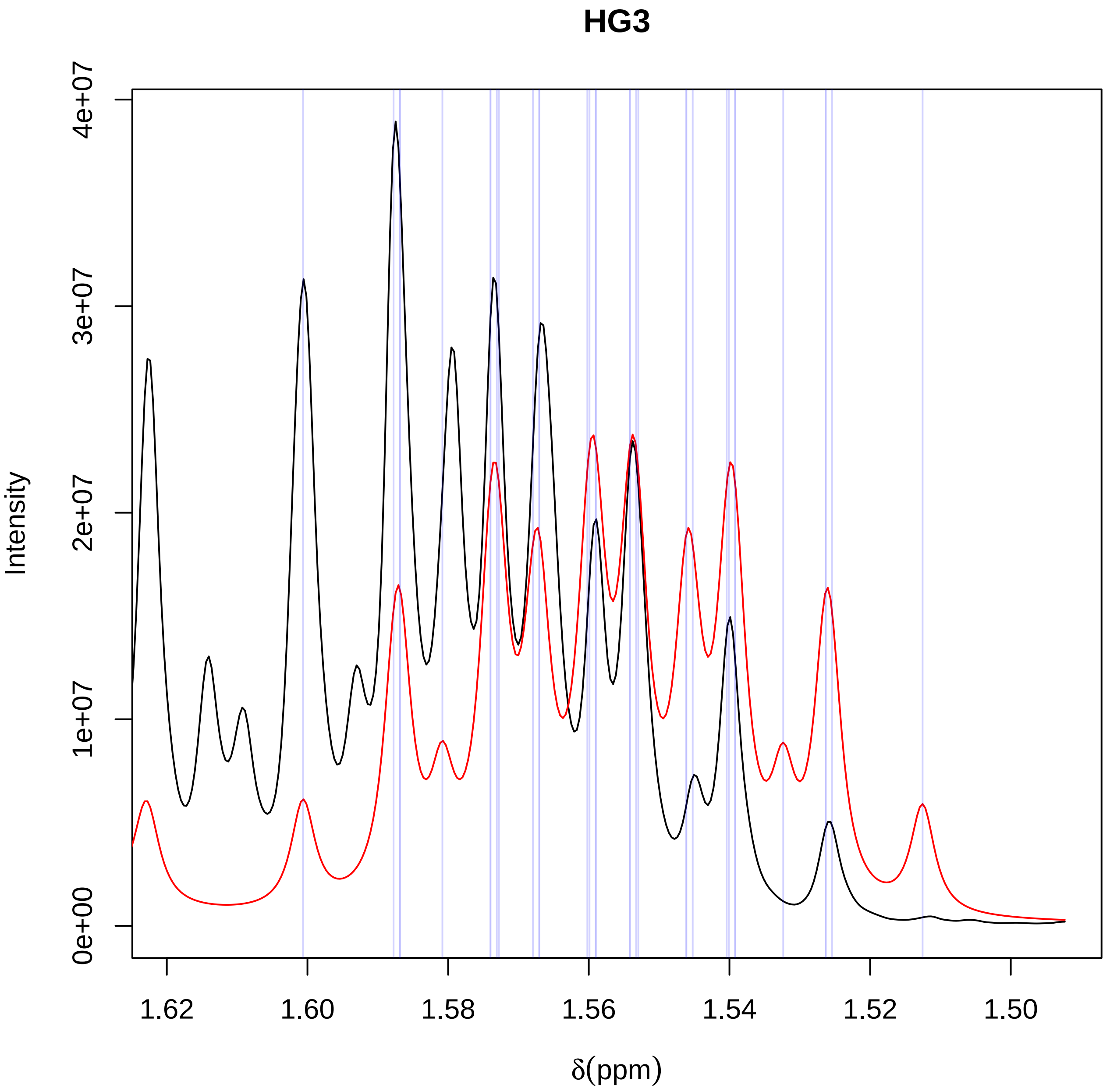

HG3
Intensity
0e+00
1e+07
2e+07
3e+07
4e+07
1.62
1.60
1.58
1.56
1.54
1.52
1.50
δ(ppm)

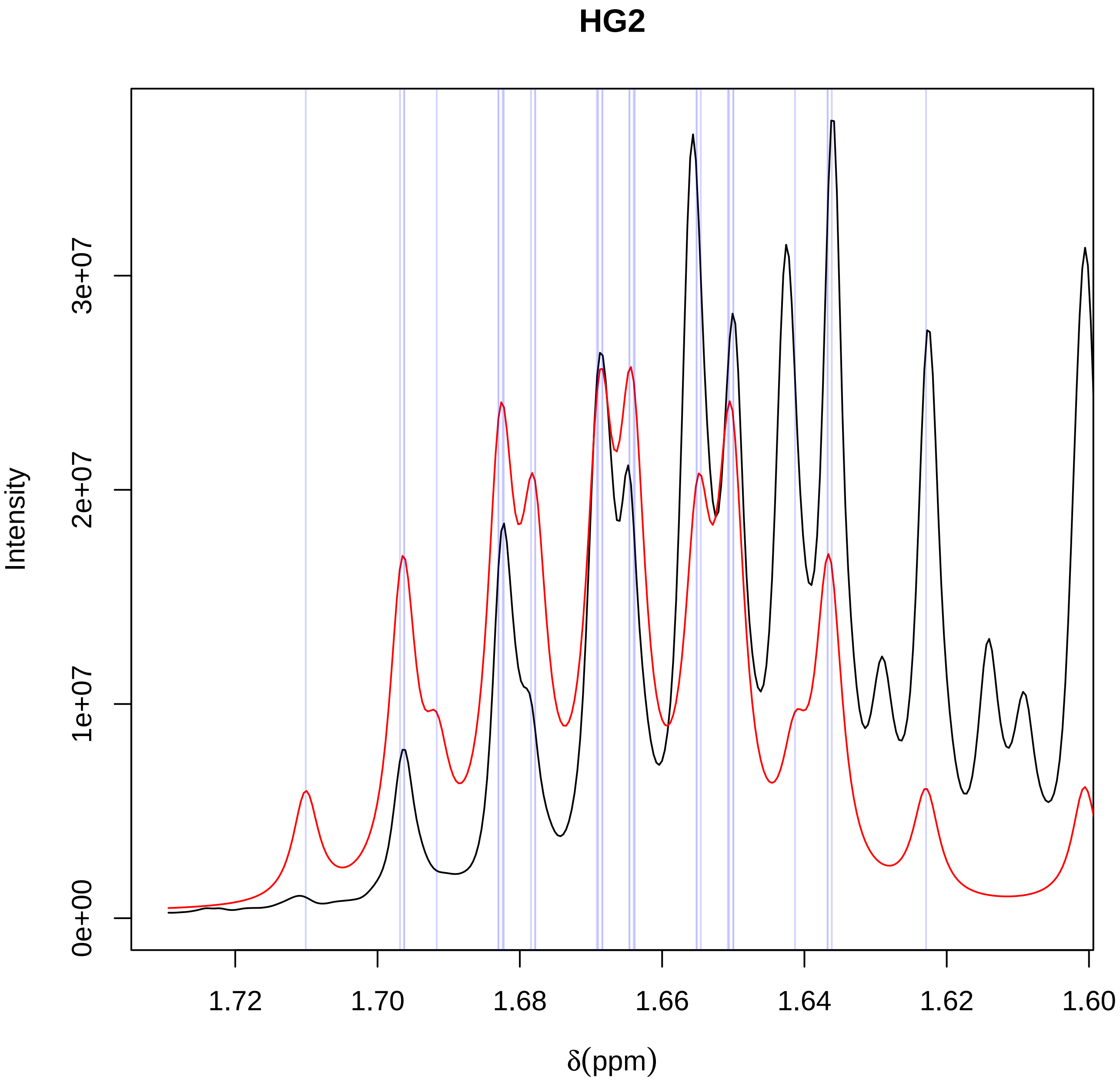

HG2
Intensity
0e+00
1e+07
2e+07
3e+07
1.72
1.70
1.68
1.66
1.64
1.62
1.60
δ(ppm)

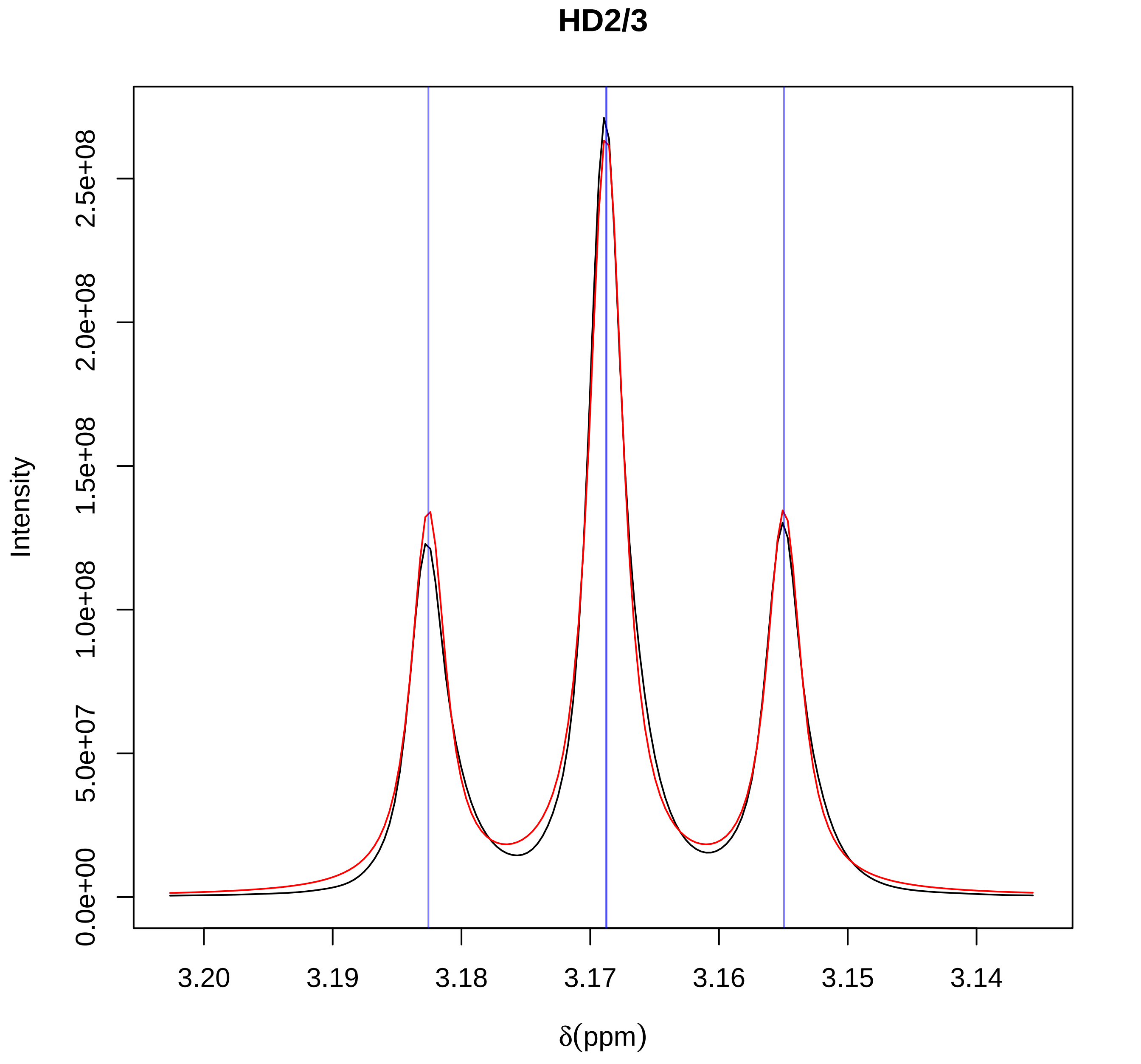

HD2/3
Intensity
0.0e+00
5.0e+07
1.0e+08
1.5e+08
2.0e+08
2.5e+08
3.20
3.19
3.18
3.17
3.16
3.15
3.14
δ(ppm)
